# Aims and tasks in parental caregiving for children receiving palliative care at home: a qualitative study

**DOI:** 10.1007/s00431-016-2842-3

**Published:** 2017-01-11

**Authors:** Lisa M. Verberne, Marijke C. Kars, Antoinette Y. N. Schouten-van Meeteren, Diederik K. Bosman, Derk A. Colenbrander, Martha A. Grootenhuis, Johannes J. M. van Delden

**Affiliations:** 1 Department of Medical Humanities, Julius Center for Health Sciences and Primary Care, University Medical Center Utrecht, Heidelberglaan 100, 3508 GA Utrecht, The Netherlands; 2 Department of Pediatric Oncology, Emma Children’s Hospital, Academic Medical Center, Meibergdreef 9, 1105 AZ Amsterdam, The Netherlands; 3 Department of Pediatrics, Emma Children’s Hospital, Academic Medical Center, Meibergdreef 9, 1105 AZ Amsterdam, The Netherlands; 4 Psychosocial Department, Emma Children’s Hospital, Academic Medical Center, Meibergdreef 9, 1105 AZ Amsterdam, The Netherlands; 5 Princess Máxima Center for Pediatric Oncology, Lundlaan 6, 3584 AE Utrecht, The Netherlands

**Keywords:** Family adjustment, Parental caregiving, Parenting, Paediatric palliative care, Home care

## Abstract

In paediatric palliative care (PPC), parents are confronted with increasing caregiving demands. More children are cared for at home, and the need for PPC of children is lengthened due to technical and medical improvements. Therefore, a clear understanding of the content of parental caregiving in PPC becomes increasingly important. The objective is to gain insight into parental caregiving based on the lived experience of parents with a child with a life-limiting disease. An interpretative qualitative study using thematic analysis was performed. Single or repeated interviews were undertaken with 42 parents of 24 children with a malignant or non-malignant disease, receiving PPC. Based on their ambition to be a ‘good parent’, parents caring for a child with a life-limiting disease strived for three aims: controlled symptoms and controlled disease, a life worth living for their ill child and family balance. These aims resulted in four tasks that parents performed: providing basic and complex care, organising good quality care and treatment, making sound decisions while managing risks and organising a good family life.

*Conclusion:* Parents need early explanation from professionals about balancing between their aims and the related tasks to get a grip on their situation and to prevent becoming overburdened.

**Table:** 

**What is Known:** • *In paediatric palliative care*, *parents are confronted with increasing caregiving demands*. • *Parenting is often approached from the perspective of stress*.
**What is New:** • *Parents strive for three aims: controlled symptoms and controlled disease, a life worth living for their child and family balance*. • *Parents perform four tasks: providing basic and complex care, organising good quality care, making decisions while managing risks and organising a good family life*. • *Professionals need insight into the parents’ aims and tasks from the parental perspective to strengthen parents’ resilience*.

**Electronic supplementary material:**

The online version of this article (doi:10.1007/s00431-016-2842-3) contains peer-reviewed but unedited supplementary material, which is available to authorised users.

## Introduction

In paediatric palliative care (PPC), most seriously ill children are predominantly cared for at home [18, 31, 43]. Therefore, parents of a child with a life-limiting disease (LLD) are confronted with increased caregiving demands, and also have to cope with the inevitability of a premature death of their child [12]. The spectrum of LLDs requiring palliative care during childhood is broad and heterogeneous. LLDs are generally divided into four categories (Table 1) [1]. The duration of PPC and the needs of these children vary widely among the categories.

**Table 1:** Categories of life-limiting diseases [1]

Category	Description	Examples
Category 1	Life-threatening conditions for which curative treatment may be feasible but can fail. Where access to palliative care services may be necessary when treatment fails or during acute crisis, irrespective of the duration of that threat to life	Cancer; irreversible organ failure of heart, liver or kidney
Category 2	Conditions where premature death is inevitable, where there may be long periods of intensive treatment aimed at prolonging life and allowing participation in normal childhood activities	Cystic fibrosis; muscular dystrophy
Category 3	Progressive conditions without curative treatment options, in which treatment is exclusively palliative and may commonly extend over many years	Batten’s disease; mucopolysaccharidoses
Category 4	Irreversible but non-progressive conditions causing severe disability, leading to susceptibility to health complications, and likelihood of premature death	Severe cerebral palsy; multiple disabilities such as following brain or spinal cord injury

Because PPC is a relatively young specialty, current knowledge on parental caregiving mainly relies on studies in chronically ill children, not facing life-limiting issues of their disease and in children treated for cancer. It shows that the parenting role intensifies and expands beyond routine physical care [21, 33, 38, 44, 48]. This expanded parenting role includes nursing, technical and emotional tasks, such as providing childcare, learning about the disease and its treatment options, managing their child’s disease, organising all aspects of their child’s daily life and care and managing their own particular situation [4, 11, 12, 21, 39, 44, 47, 48].

Studies on parental caregiving in PPC are mainly performed in paediatric oncology and focus on the end-of-life (EOL). Besides the expansion of caregiving tasks, these studies show that parents have to deal with uncertainty and to adapt to an accumulation of losses related to their child’s physical and functional decline [6, 14, 26]. Although parents intend to act in their child’s best interest, including a good death, many of them struggle with facing reality and the timely transition from preserving their child at all costs towards being prepared to let their child die [2, 10, 14, 16, 23]. Moreover, parents emphasise they have to ‘navigate uncharted territory’ and lack professional guidance, resulting in feelings of isolation and abandonment [15, 37]. A recent review on chronic care situations in children showed the discrepancy between the parental learning needs and the information provided by healthcare professionals (HCPs), stressing the necessity to elicit the parents’ perspectives and to take the families’ complete situation into account [30].

Paediatric illnesses or injuries affect many children and parents because they are often brought into healthcare settings under adverse and often life-threatening circumstances [20, 32]. These circumstances concern potentially traumatic medical experiences that might lead to stress responses [20]. As such, parenting a seriously ill child is often approached from the perspective of stress [38, 48], as is represented in the paediatric medical traumatic stress model [20, 32]. In PPC, parental caregiving is also considered distressing and potentially traumatic for parents [25, 32, 36], since living towards a child’s death understandably causes disruption and grief [12]. Studies indicate that parents of children with a LLD, who perceive a high risk of life threat and complications, are at increased risk of post-traumatic stress disorder (PTSD) [19, 32]. While HCPs cannot protect parents from such risks, they should however try to strengthen the parents’ resilience and prevent distress as much as possible [12, 34, 35]. This starts with an understanding of parental caregiving from the parents’ perspective. In addition, since driven by technical and medical improvements PPC may last over many years, a clear understanding of the content of parental caregiving in PPC from the parents’ perspective becomes increasingly important. Therefore, the aim of this article is to provide a generic and comprehensive overview of parental caregiving, based on the lived experience of parents caring for a child with a LLD.

## Methods

To elucidate the parents’ perspective, we conducted an interpretative qualitative interview study using an inductive thematic analysis [3, 8, 40]. This study was part of a larger study to evaluate a pilot with a paediatric palliative care team (PPCT; Box 1). The focus of this article is to provide insight into parental caregiving in PPC from the parents’ perspective. The role of the PPCT from the parents’ perspective will be described in a separate article.

**Table:** Box 1 Description of the paediatric palliative care team (PPCT)

In June 2012, the first Dutch PPCT was initiated as a 3-year pilot project at the Emma Children’s Hospital in Amsterdam. The multidisciplinary PPCT consists of five specialised paediatric nurses trained and experienced in PPC, two child life specialists, a psychologist, a social worker and a chaplain. Additionally, two paediatric oncologists and two paediatricians are committed for regular consultation. The PPCT is responsible for the coordination, continuity and quality of PPC, irrespective of the child’s place of residence. They strive to avoid acute demands for support by a proactive attitude. The support provided by the PPCT is continuous throughout the disease trajectory, including a 24-h availability and bereavement care. The PPCT bridges the gaps between home and hospital and navigates parents through the complex care processes by regular contact through phone calls, e-mails, and personal visits at home and during hospitalisations. In addition, the PPCT strengthens regular care at home by educating and coaching the other healthcare professionals involved. If regular care fails, the PPCT is competent and qualified to take over the care by providing temporary nursing care at home. For the possibility to discuss patients, maximum exchange of palliative care knowledge and optimal deployment and collaboration between team members, the PPCT has weekly multidisciplinary conferences.	

### Sample

A purposive sample of Dutch-speaking parents of children with a LLD primarily residing at home who were referred to the PPCT from a Dutch university children’s hospital (Emma Children’s Hospital, Amsterdam) was included. Referral to the PPCT ensured a general agreement among HCPs that PPC was indicated and thus provided access to families of children with a variety of diseases, who could maximally inform us about parental caregiving in PPC [28]. To capture a wide range of perspectives, variation in selected children was sought with respect to malignant (MD) and non-malignant diagnoses (NMD) and phase of the disease trajectory that increases the need for PPC: the palliative trajectory. Based on literature, four phases of the palliative trajectory were distinguished: diagnostic phase, phase of loss of normality (adjusting to new normality), phase of decline and the dying phase [15, 45]. Parents of 35 cases were identified as eligible. A member of the PPCT or the treating physician introduced this study to the parents and asked permission for the researchers to contact them. In six identified cases, the introducing HCP considered the parents’ situation too vulnerable to inform them about the study. Parents of 29 children were invited by telephone to participate by the researchers. In five cases, parents refused participation. Reasons for refusal were as follows: no time (*n* = 2), too burdensome (*n* = 2) and unknown reason (*n* = 1). In total, 24 mothers and 18 fathers of 24 children were interviewed. For patient characteristics, see Table 2. In three cases, parents (*n* = 6) were intentionally approached to participate after the child’s death, and in three cases, a second interview after the child’s death was performed with five parents to gain deeper insight into parental caregiving during the end-of-life and dying phase.

**Table 2:** Characteristics of the parents (*n* = 42) and their ill child (*n* = 24)

Characteristics	Number (*N*)	Percentages (%)
Gender parent		
Male	18	43
Female	24	57
Age of parent^a^		
<30	2	5
30–40	29	73
>40	9	23
Marital stage		
Married/cohabiting	38	90
Divorced/not cohabiting	4	10
Education		
Low^b^	5	12
Middle^c^	15	36
High^d^	22	52
Age of child at first interview		
0–1	1^e^	4^e^
1–5	13^f^	54^f^
5–12	7	29
12–16	2	8
≥16	1	4
Child gender		
Male	12	50
Female	12	50
Child diagnosis		
Non-malignant disease (total)	15	63
Congenital anomalies	11	46
Neurodegenerative disease	2	8
Metabolic disease	2	8
Malignant disease (total)	9	38
Central nervous system tumour	5	21
Bone/soft tissue sarcoma	2	8
Neuroblastoma	1	4
Leukaemia	1	4
Time since diagnosis		
0–6 months	2	8
6–12 months	3	13
1–2 years	7	29
2–5 years	8	33
> 5 years	4	17
Palliative phase at first interview		
Diagnostic phase	0	0
Phase of loss of normality	15	63
Phase of decline	6	25
Dying phase	3	13
Siblings per case		
0	5	21
1	11	46
2	7	29
3	1	4

Percentages may not equal 100 due to rounding

^a^Age of two parents is missing

^b^Low: primary school, lower secondary general education, lower vocational education

^c^Middle: higher secondary general education, intermediate vocational education

^d^High: higher vocational education, university

^e^In one case, the interview took place after the child’s death

^f^In two cases, the interview took place after the child’s death

### Data collection

In total, 47 individual open interviews took place at home. The interviews were held between August 2013 and November 2015 and lasted from 30 min to 2 h. The interviewers (LV, MK, MB) were independent researchers from a different university from where the PPCT is seated. A topic list (Supplement 1) based on literature and experts’ knowledge was used to guide the interviews. Topics relevant for this study were parenting, parental caregiving, care facilities, parents’ life, self-efficacy and family life. Interviews were audio recorded and transcribed verbatim. The study was approved by the research ethics committee of the Academic Medical Centre Amsterdam (June 12, 2013; Reference number: W13_120 no. 13.17.0153). Written informed consent was obtained from all participating parents.

### Data analysis

An inductive thematic analysis was used [3, 8, 40] in accordance with methods that optimise validity and rigour [29]. During the entire process, three researchers (LV, MK, JvD) supported by a research assistant were involved. They used joint meetings to reach agreement on interpretation of the data and findings and worked towards consensus. Therefore, researcher triangulation was ensured to improve reliability and validity of the analysis.

The thematic analysis consisted of three phases. Firstly, the researchers (re)read the transcripts of eight interviews individually to get familiar with common aspects and phrases in the context of the interview [3, 8, 40]. At least two researchers analysed and initially coded with paper and pencil the eight transcripts individually and compared interpretations together. The meaning of the separate text fragments was determined by interpreting them in light of the whole interview [8, 22]. The initial codes were recoded, resulting in an adapted code list with themes and concepts at a more abstract and conceptual level [8].

During the second phase, every new interview was read and discussed by at least two researchers. One researcher (LV) and a trained research assistant coded all transcripts, supported by the software program NVivo10. After coding each transcript, the code tree was evaluated and, if necessary, revised. The different codes were sorted into potential themes, which were defined and refined [3]. To guide constant comparison, the research team went back and forth between the different steps and the entire dataset to capture the key aspects of the themes in the raw transcripts.

Thirdly, the researchers identified the relationship between the themes [3] and integrated the themes into a descriptive model [40]. To ensure validity and provide transparency of the results, an audit trail was made by the core members of the research team (LV, MK, JvD), in consultation with the other researchers involved in this study, to record methodological choices and substantive ideas and concepts related to the interpretation of the data. Saturation was reached on a conceptual level. The results were externally validated by two parent association representatives, respectively for children with malignant and non-malignant diseases. In addition, we checked our findings in an expert meeting among nine HCPs experienced in paediatrics, PPC and/or homecare. This has not led to adjustments of the results.

## Results

### Being a ‘good parent’

**Fig. 1:**
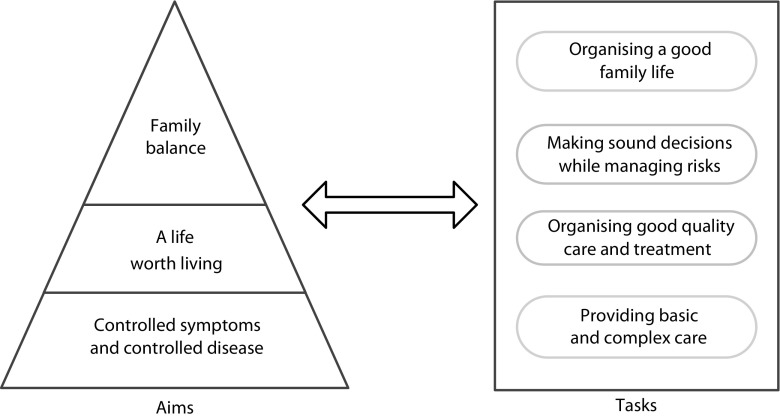
Aims and tasks of parents caring for a child with a LLD

All parents expressed their ambition to be a good parent for their child in the extraordinary situation of knowing that their child’s life is limited and within an unknown time span where they will have to direct their child’s EOL. In response to the perceived vulnerability, the disease-related suffering and the efforts their child had to make due to his/her illness, parents desired to be the best parents their child could wish for. The wish to be a good parent became manifest in three aims parents consciously or unconsciously strived for, as identified from their narratives. In addition, four groups of tasks connected to the aims were identified (Fig. 1). First, the aims and the way parents struggled to achieve the aims are described. Then, the related parental tasks are presented. Representative quotations were chosen to illustrate the identified aims and tasks (Tables 3 and 4).

**Table 3:** Quotes that illustrate the parents’ wish to be a ‘good parent’ and the parents’ aims in the care for their child with a LLD, chosen from eight interviews with parents

Theme	Quote
Being a good parent	Case 7: boy, 9 years, NMD. Father: *When he dies, if he could think, this is the way it had to be, it sucks that I had this disease but I could not have had better parents with this disease, to go this route with me. That should be his conclusion on the last day.* Case 23: boy, 4 years, MD. Father: *It takes a lot of energy to have such a child, besides that the care takes a lot of energy. You are going to think about many things, whether you like it or not. Do I mind it? No, I do not mind it because he deserves everything, he deserves the best, so I do everything for him. Yes, we give him whatever we can give him. It just takes a lot of energy, that’s all. So we are exhausted when we go to sleep in the evening, you are exhausted. But I do not mind.*
Controlled symptoms and controlled disease	Case 21: girl, 3 years, NMD. Mother: *You also want her not to be sick too often, that she has energy, because that was a problem for a long time. For that reason she started the night ventilation. And you see she has more energy and feels good and she has less pneumonia and lower respiratory tract infection, and that was absolutely the main goal. And that remains the goal, to have her as well as possible and that she can enjoy life as much as possible.*
	Case 20: girl, 6 years, MD. Mother: *Last week I contacted the doctor,* via *a telephone consultation, because I found that she was deteriorating, she started choking, drooled a lot. And her speech was worse. So since last week, in consultation with the doctor, we started the Dexamethasone again. And again today a telephone consultation with her about how it is now and whether that is enough.*
A life worth living	Case 2: girl, 4 years, NMD. Father: *My only concern is that she can have fun and enjoy herself and whether that is with a clothes pin or with a ball, having fun is the most important to me. Then this is feasible for her and then it is ok.*
	Case 23: boy, 4 years, MD. Mother: *Yes, that he has time for as long as possible, and can be light-hearted and have fun, enjoy himself, enjoy being together. That he can enjoy the little things. That we can do the things that give him energy. And yeah, that he is having a good time.*
Family balance	Case 14: girl, 1 year, NMD. Father: *Since the birth of Maaike, life has been like riding a large high-speed train going 400 km an hour. And it is very difficult to relax and be there for each other. Look, the most important thing is Maaike and her well-being, but we also have to be there for each other, so Machteld (mother) and I have to work to maintain our relationship, otherwise it will go wrong. But right now being there for each other is proving very difficult. And we both know that we have to do that… but well, Maaike is currently recovering from an illness, and we currently do not dare to leave her in other people’s care. So the decision is very simple, you choose Maaike. But we do know that it’s very important to go out for an evening, to go out to dinner together, to be with each other. But yeah, right now that’s not possible.*
	Case 13: boy, 5 years, MD. Mother: talking about the time that her son received chemotherapy: *It was just very tough for everyone, also for Jayden (sibling, 6 years), because he had to miss us very often because he was with his grandparents. Yeah and we do not want that anymore. […] Jayden, he has to be ok, he has to come out as unscathed as possible, to come through it. To some extent this is not possible, but you need to support him in the best possible way.*

Some quotes are slightly modified to improve readability. Names are fictitious

*MD* malignant disease, *NMD* non-malignant disease

**Table 4:** Quotes that illustrate the parents’ tasks in the care for their child with a LLD, chosen from eight interviews with parents

Theme	Quote
Providing basic and complex care	Case 21: girl, 3 years, NMD. Mother: *We are busy with everyday things, because she cannot do them herself. You have to make sure she sits one moment and lies down the next. PEG-tube care, make sure her lungs do not fill up, suctioning. Oxygen. And on and on. And also with her bowel movements, that that all goes well. But also that she develops in her own way, so you are also occupied with the standing-table or working her muscles, but also doing something creative with her. It’s is always a lot. But it is something that you are already very used to. And, of course, providing medication, that’s what you start with, and tube feeding. That kind of stuff I’m actually doing now as part of her daily care.*
	Case 17: girl, 9 years, MD. Father: *The only kind of care that we have now is the home care; they will replace the tube if Tessa’s tube has fallen out, otherwise we do everything ourselves. Administering the chemotherapy, which is easy, and the medication; that’s all easy, otherwise she doesn’t really have any facilitated care, no.*
Organising good quality care and treatment	Case 6: boy, 2 years, NMD. Mother: *There are so many things you have to think about, and that takes a lot of energy. You have to give direction to all the caregivers. And ‘PGB’ (resources) and the home care organisation, you’re also busy with them and then there is a hearing and what not. There is always something. And usually such bureaucratic matters are not settled at once. So it takes you a great deal of time to arrange everything, to make clear to people what you mean and to fill out a lot of forms or call a lot of people again and again to say please hurry up… It really is a kind of full-time job to arrange it all successfully.*
	Case 22: girl, 6 years, MD. Mother: *Then we were gathering information, exploring this and that… and we discovered that the irradiation method with protons for children with brain tumours did not yet exist in the Netherlands. However, it did exist in Germany, France, Spain, Switzerland. [..] So we talked about it with the hospital. After much humming and hawing we went to Germany and did the irradiation there. We were so happy that we chose to do it there, and it didn’t bother her at all.*
Making sound decisions while managing risks	Case 8: boy, 6 years, NMD. Father: *We don’t use day care or other child care. And that I think has to do with two reasons, firstly because Linda (mother) is very happy to be with him. And she likes to keep him close to her. And that when I am at work I have peace of mind because he is with Linda when I’m not there. And secondly because we are afraid that if he, for example, were picked up by a taxi and went to school every day, the chance of his getting a respiratory infection would absolutely be increased and that is the second reason not to do that at this time.*
	Case 23: boy, 4 years, MD. Father: *You have to make a number of decisions very soon. Which road are you taking, which one you’re not. [...] It goes very quickly. You get very little time to think calmly, you don’t have time to think, you have to make choices. Yes, I found it difficult. But there is no other way. We were told there are three choices, you do nothing, you take the treatment in one particular university hospital or in this university hospital. And we were told all the pros and cons. And we finally said, ok, tomorrow we will call and let you know our choice. And we chose quality of life and quality for our son, that he had the least pain, so we opted for the short-term treatment, and we did not chose science.*
Organising a good family balance	Case 6: boy, 2 years, NMD. Father: *That is really divided. At home, we do everything together, but if he gets sick, I go to the hospital with him. My wife stays with the children and takes care of everything at home and comes to the hospital every other day with one of the children or alone. And if he is really doing poorly, then she comes every day. But we need to create a bit of peace and quiet at home, with the other children.*
	Case 17: girl, 9 years old, MD. Mother: *And the difficulty with Tessa is, you have to explain everything, why do I have to take this and why do I get that? But on the other hand she pulls us through it each time, like okay…we have to… And also the other two, the twins, have to go to school, so luckily we still have our structure to some extent… meals on time, on time to bed. So there is a rhythm. We try to keep to that as much as possible. At half past six it should be quiet here. Then they have to sleep. Then mom and dad also have some time together.*

Some quotes are slightly modified to improve readability. Names are fictitious

*MD* malignant disease, *NMD* non-malignant disease

### Aims

The parents’ aims were (1) controlled symptoms and controlled disease, (2) a life worth living and (3) family balance.

#### Controlled symptoms and controlled disease

All parents described that it was of major importance for them to reach controlled symptoms and controlled disease of their ill child. They mentioned that controlling symptoms was of major relevance for maximal comfort for their child or at least that inconveniences were minimised as much as possible. Additionally, they mentioned that controlling the disease was a matter of preventing loss of their child or maximally prolonging their child’s life. At some point during the disease process, parents were informed by HCPs that cure from the disease or preserving life of their child was no longer possible. Despite this information, many parents, both from the MD and NMD group, still had the ambition to find possible treatments to control their child’s disease. Therefore, parents constantly worked to reduce, relieve or prevent symptoms of the disease and side effects of the treatment and also searched for optimal treatment to prevent further progression of the disease. When parents believed that controlling the disease was no longer a realistic aim, their focus shifted towards merely comfort care, whereby symptom control remained important.

#### A life worth living

Despite their focus on controlled symptoms and controlled disease, parents emphasised seeing their child as a beloved person who deserves a life worth living for the time that is left. They wanted their child to have fun, enjoy his/her life and make as much out of his/her life as possible. Most parents felt challenged to create a life worth living for their child. Especially when their child had limited abilities or when their child deteriorated and lost his/her abilities for life fulfilment, parents put even more efforts towards creating a life worth living.

#### Family balance

Parents aimed a variety of aspects that could be categorised as family balance. Family balance is a situation in which all individual family members can keep going, experience well-being and are able to develop within their full potential. It also contains established parental responsibilities, such as earning an income or organising a holiday.

Parents felt challenged in achieving this aim because they continuously had to adapt to the demanding care situation and to rearrange family life, while meeting the needs and interests of all family members involved. They mentioned that they had to deal with the limited flexibility in daily life, the disruption of the self-evident togetherness of their family and the siblings’ need for attention. Most parents could not immediately leave home for spontaneous or unexpected activities because they needed more time in advance to schedule and prepare activities than ‘healthy’ families. Planned activities often needed cancelling, for instance when symptoms of their child suddenly worsened. Due to the extensive involvement in childcare and the intensive use of healthcare facilities, including hospitalisations, the opportunity to be together as a family was limited. Parents felt forced to give priority to the needs of their ill child, thus the siblings often came in second place. Many parents noticed that siblings received less attention and often had to wait until they had finished the care, which they could not postpone, for their ill child. Parents felt uncomfortable about this situation but often lacked the opportunity to act otherwise given the urgency of the needs of their ill child.

### Struggling to achieve all aims

From the parents’ stories, it could be concluded that they became aware of the three aims over time. In addition, they experienced difficulties in balancing the aims because they tried to achieve the three aims at the same time.

#### Becoming aware of the aims

Parents mentioned how, initially, they were fully occupied by the aim of controlled symptoms and controlled disease as first priority. Parents emphasised that they, in their perspective, had learnt how to control the symptoms and to limit the burden and progression of the disease and how to respond to their child’s care needs. During this learning process, many parents had felt thrown back on their own. However, they became familiar with the disease, the treatment options, their child’s special needs and preferences, the healthcare system and what the new world of their child entailed. Most parents had developed their ability to assess, decide on and perform all disease-related tasks; however, some parents continued to struggle with the complexity of achieving controlled symptoms and controlled disease and felt uncertain, particularly when the disease progressed or when complications occurred.

For parents, the focus on creating a life worth living for their child was boosted once the LLD was diagnosed or, in MD, when it became clear for parents that treating cancer was no longer considered to be effective. From the start of their child’s disease, most parents intuitively felt the importance of a family balance. However, they accepted the disrupted family balance because their first and second aim of controlling symptoms and controlling disease and living a meaningful life had priority. Family balance obtained a clearer focus when the disease trajectory lasted longer and when the disease and symptom management and the child’s well-being were at a manageable level.

#### Balancing the aims

In the context of their child’s inevitable death, parents wanted to do everything as well as possible and tried to maximise all separate aims. However, they experienced that the efforts for creating a life worth living for their ill child and achieving a family balance were easily overruled by the efforts for controlling symptoms and, if possible, controlling disease, because the child’s symptoms or disease always intruded to the foreground. Consequently, controlled symptoms and controlled disease appeared to stay the predominant aim for parents.

A life worth living for their ill child was the second dominant aim. Parents mainly succeeded herein when they, in their perspective, had controlled the symptoms and, if possible, the disease. Only when their child’s death was near, some parents ignored their first aim in order to create a life worth living. For example, while their child had pain and wanted to play with friends, parents decided to delay the start of pain medication in order to enable their child to experience life fulfilment instead of being asleep as a side effect of the medication.

Achieving the first and second aim was a prerequisite to work towards a family balance. Therefore, many parents described their family balance as fragile, as it was rapidly disturbed by an increase of the symptoms, progression of the disease or a decrease of the child’s well-being. In these situations, the aim for a family balance was easily overruled by the parents’ need to control the symptoms and, if still realistic, to control the disease and by their ideal of a meaningful life.

Because parents tried to achieve all three aims, they had to keep several balls in the air at the same time. Some parents became aware of the necessity to balance between the aims, were able to develop themselves herein and increasingly took direction to achieve all three aims. For example, some parents realised that they also needed to give attention to their partner, other children and/or friends; otherwise, all these relations would be lost after their child’s death. Other parents felt overwhelmed by the multiplicity and complexity of the first aim and were not able to look beyond controlling their child’s symptoms and disease.

### Tasks

With maximal commitment, parents performed many intertwined tasks, originating from the child’s disease and the abovementioned aims. Four groups of tasks were identified: (1) providing basic and complex care, (2) organising good quality care and treatment, (3) making sound decisions while managing risks and (4) organising a good family life. The accomplishment of the tasks by parents determined the level of achievement of their aims, varying per family and child.

#### Providing basic and complex care

For many parents, the caregiving tasks to achieve controlled symptoms and controlled disease and to create a life worth living were unavoidable and numerous. The caregiving tasks consisted of assisting in the child’s activities of daily living (ADL), symptom management, medical technical procedures, offering sleep support, supporting well-being and creating life fulfilment for their child. Many parents described how they learnt to provide complex medical care, such as preparing and providing medication, suctioning, giving tube feeding or fixing a prosthesis. These procedures needed to be attuned to their child’s needs, abilities, coping strategies and learned routines and rituals to help him/her to accept undergoing the procedures of which some were life sustaining.

All parents felt the need and took their responsibility to monitor the child’s physical condition and well-being and to intervene adequately when needed. Consequently, parents had limited control over their efforts for controlled symptoms and controlled disease, including how these efforts influenced their daily life. Besides this responsibility, parents emphasised that they considered themselves as being the best carers because they cared with parental love attuned to their child’s needs. Therefore, they found it difficult to leave (complex) care to others, such as HCPs or informal carers. Many parents mentioned that even when they were supported by homecare nurses or during hospitalisations, they still provided many components of care. Additionally, when HCPs or informal carers became involved, parents had to guide them towards providing the care for their child attuned to his/her needs.

The intensity of providing basic and complex care largely varied per family and child and throughout the disease trajectory. Some parents could not lose their child out of sight to prevent the risk of severe life-threatening situations, which resulted in 24-h per day caring for their child, whereas others provided mainly routine childcare and facilitated their child to attend school.

#### Organising good quality care and treatment

Many parents emphasised how they had searched for the best treatment and professional support. They coordinated care and care facilities and arranged many practical things, such as equipment, reimbursements and medication at home. Some parents also arranged things, such as a special computer or toys, to enable their child to communicate and to develop, which potentially improved the child’s well-being. Over time, some parents arranged (more) homecare to have some respite in favour of having time for the siblings or themselves (family balance).

Because parents felt final responsible for their child and family, they primarily performed this task. Many parents described this task as time-consuming and difficult because it was an ongoing process, even when their child was stable, and it never succeeded at once. Also, many actors and organisations were involved in creating well-organised childcare, making this task even more complex. Moreover, parents mentioned that this task required many efforts and valuable time, which they preferred to spend with their child. Especially when their child’s death was near, parents wanted to enjoy the time left together with their child and not being busy with arranging care and practical things.

#### Making sound decisions while managing risks

Parents described that they made numerous, both minor and major, decisions in daily life aimed at maximising accomplishment of the three aims. For example, “is it safe enough to let my 15-year-old son take a shower by himself despite the risk of falling due to his amputated leg?” Because some parents, for instance, decided to accept day care to gain time for the siblings or to let their child attend school because he/she did enjoy this, their ability to control the disease and symptoms according to their own standards could decrease. Therefore, many parents often felt forced to weigh arguments, consider alternatives and make decisions to achieve a justifiable balance between their aims.

Especially, making decisions entailed weighing the major risks of losing their child. Parents explained how they decided to maximally protect their child by reducing the risk of worsening the symptoms and/or the disease or provoking a life-threatening situation. This often resulted in protective behaviour that increased their workload. For example, some parents avoided day care to minimise the risk of infection. At other occasions, parents consciously chose not to emphasise the control of symptoms and control of disease and accepted a possible risk of deterioration in favour of a life worth living or a good family balance.

Because parents felt responsible for making the right decisions, they often felt tensions, particularly when they had to make decisions that allowed EOL to come or when they had to choose the least bad option. For instance, when new symptoms appeared, they had to choose between accepting the increase of the symptom load of their child or adapting medication running the risk of new or increased side effects. Many parents told that by moments, they felt overwhelmed by the large amount of decisions they had to make and felt overruled by the type and the impact of decisions and the short period of time they had available for making these far-reaching decisions. Some parents mentioned they had to negotiate for sufficient time until they felt comfortable to make a sound decision.

#### Organising a good family life

Most parents described that having a child with a LLD affected all family members. In favour of a family balance, they tried to integrate their extraordinary situation in daily life. They supported their child to attach to normal life or embedded their situation in a life as normal as possible. To relieve their workload, many parents chose to divide the caregiving tasks and the responsibility of earning an income. This could result in feelings of shortcoming and having lost relevant aspects of personal development, for one or both parents.

Many parents described they discovered in particular that rhythm and routines were helping in integrating caregiving in daily life. Rhythm took form of a daily pattern, providing guidance in the things that had to be done, such as wake up in time, providing childcare, cooking, housekeeping and bringing the children to school. Routines were tasks where they no longer had to think about and which became part of their pattern, for example how to prepare medication or when to give tube feeding. Parents told that by applying family rhythm and childcare routines, they gained time and energy for other activities.

When parents were aware of their aim for a family balance, they continuously had to weigh the needs of the individual family members. Sometimes, this meant that they had to reorganise care in favour of the family balance. For example, parents decided that when their child would be hospitalised again, one of them stayed with their child and one of them stayed at home with the siblings to pay them proper parental attention and stay connected. This choice came instead of staying in the hospital of both parents and letting the siblings stay at their grandparents as they did before.

## Discussion

We identified that, based on their ambition to be a good parent in a situation where parents tried to prevent child loss and had to direct child loss in the end, parents strived for three main aims in caring for their child with a LLD. Parents primarily aimed for optimal controlled symptoms and controlled disease. Over time, the aims of a life worth living and a family balance gained importance. Since the time with their child is finite, parents developed a major need to concurrently achieve each separate aim and felt under pressure because everything had to be as good as possible. However, they could not always succeed herein, resulting in considerable distress for parents. To achieve the three aims, parents performed four interconnected tasks: providing basic and complex care, organising good quality care and treatment, making sound decisions while managing risks and organising a good family life. These tasks were relentless for parents because the accomplishment of the tasks determined the level of achievement of the aims. Although MD and NMD and their disease trajectories differed, the aims and tasks as experienced by parents in both groups were quite similar.

From this study, it follows that being a good parent included not only maintaining their child’s health and ensuring that their child had a good life, which is earlier described [10, 24, 46, 47] but also achieving a situation in which all family members could keep going and experienced well-being. This study adds that the parents’ aims not only guided parental caregiving but also encouraged them to leave no stone unturned to achieve all these aims, particularly controlled symptoms and controlled disease and a life worth living. As such, many parents were absolutely involved in childcare and ran the risk of a suppressed family life. In addition, the pressure for parents accumulated by doing the utmost in a limited time while also creating a rewarding time. These aspects contributed, together with the threat of future child loss, to the parents’ distress and could be an explanation for the increased risk of a PTSD [19, 32].

Some of the identified parental tasks are earlier reported but not yet in a comprehensive overview within the context of PPC as was done in this study. Like others, we found that parents felt largely responsible to provide childcare by themselves no matter how complex it is [4, 6, 11, 12, 44, 47, 48] and did everything to organise the best quality of care and treatment for their child. Parents also organised a good family life, which was creating time for themselves and their partner and, consistent with earlier research, maintaining any sense of normal life [6] and emotionally supporting their child and the siblings [4, 21, 27, 39]. Parents felt that succeeding in their tasks largely depended on their own efforts, even when they were supported by HCPs. However, their wish to be a good parent was a powerful internal motivator to conduct all tasks, as was till now only indicated in paediatric oncology [10, 13].

Many parents had felt abandoned and had difficulty in searching for the diagnosis and the best treatment, and in developing parental caregiving, which is in line with studies addressing parents’ role to ’navigate uncharted territory’ [15, 37, 47]. Parents mentioned this as exhausting and stressful at times, as was also described by Woodgate et al. [47]. It was seen that parents became experts in their child’s care attuned to his/her needs; however, many of them (had) felt disrupted and sometimes powerless to improve their child’s and own situation. Parents felt tensions in searching for support on the one hand and the burden of this support on the other hand, due to the limited time left with their child and the risk of a rapid disruption of their situation. Parents wanted to be there for their child and strived for preserving their parenting role and relationship with their child. Consequently, consistent with previous studies, parents found it difficult to entrust the care to informal caregivers or HCPs who, in their perception, may lack the ability to provide care at the same level as they do [6, 45, 47]. By refusing to withdraw from their caregiving tasks, some parents sacrificed their emotional and physical well-being, as previously described [6, 13].

This study showed that it is not only the complex palliative and EOL decisions but also the numerous minor decisions related to daily hassles that required attention and efforts from parents. Many parents felt inexperienced and overwhelmed to make complex and difficult EOL decisions in a limited period of time, as was also seen by Hinds et al. and Carnevale et al. [5, 10]. This study adds that parents also made many smaller decisions, whereas PPC research mainly focuses on EOL decisions [5, 7, 9, 10, 41, 42]. While making minor decisions, parents weighed the risks and the aims in daily life because every minor decision could have a major impact on controlling the symptoms and/or disease and the quality of life of their child and family. A further exploration of parental decision-making during the palliative phase could be helpful.

This study had some strengths and limitations. It was noticed that some HCPs prevented or delayed participation of eligible parents because they considered them too vulnerable or burdened, which is known as gate keeping and often seen in palliative care research [17]. This might have resulted in an underestimation of the parents’ difficulties and efforts to achieve the aims and to perform all tasks. The sample mainly consisted of native Dutch parents of one university hospital. Differing cultural and ethnic backgrounds were not captured. Nevertheless, we included both mothers and fathers and our sample showed a wide variation in diagnosis and phase of the palliative trajectory. In addition, we were able to provide the perspective of parents who currently cared for their child with a LLD. These aspects enabled us to give a realistic and comprehensive overview of parental caregiving in PPC. Our sample included a relatively large amount of children with NMD. This might have resulted in an overestimation of providing basic and complex care throughout the entire palliative trajectory because these children appeared to be more dependent on parental care for ADL than children with MD. Although Dutch people are relatively highly educated, in this study, they were overrepresented. Highly educated parents might be more capable in searching for and organising the best care for their child and might be more able to take over homecare tasks because their professional positions provided them the flexibility to do so. Therefore, in reality, the parental distress following from the aims and tasks might be even higher than seen in this study. On the other hand, it was noticed that in many families (irrespective of their education level), one parent and during the EOL phase often both parents quitted their job, which in most cases is partially financially supported by the Dutch government, enabling parents of seriously ill children to provide childcare at home.

### Implications for practice

This study shows more in-depth what parents face and how they combine parenting and caregiving. In addition to the PPC that professionals currently provide to the child and family, it could be helpful to discuss at times on a meta-perspective with parents the content of parental aims, the related tasks and the bottlenecks from both the parents’ and professionals’ perspective. This is preferably done from the start of the disease trajectory to decrease parents’ distress and to strengthen their resilience, since the awareness of their child’s LLD often overwhelms them. Based on the meta-perspective, an exploration of what is needed for parents to succeed at home can be made and organised by the parents and their homecare team together with a PPCT. Therefore, they have to define and organise the help and support in a way that is acceptable for and provides relief to parents. For example, help in the household or for other daily tasks or someone to bring a sibling to school or sports. The support should be provided by a (healthcare) professional whom parents trust and who gives active direction to the parents while at the same time not taking over their parenting role. By doing so, PPC becomes tailored to the individual needs of families in PPC and better accepted.

## Electronic supplementary material

Below is the link to the electronic supplementary material.

ESM (PDF 18.5 kb)

## Abbreviations

ADL: Activities of daily living
EOL: End-of-life
HCP: Healthcare professional
LLD: Life-limiting disease
MD: Malignant disease
NMD: Non-malignant disease
PPC: Paediatric palliative care
PPCT: Paediatric palliative care team
PTSD: Post-traumatic stress disorder
