# Supplementary material for: Aims and tasks in parental caregiving for children receiving palliative care at home: a qualitative study
Source: Eur J Pediatr. 2017 Jan 11;176(3):343–54. doi: 10.1007/s00431-016-2842-3 (PMC5321698; doi:10.1007/s00431-016-2842-3)
Supplement: Supplementary file 1 — (PDF 18.5 kb) [file 431_2016_2842_MOESM1_ESM.pdf]

**Supplement 1: Topic list**

**What is the lived experience of parents caring for a child with a life-limiting or life-threatening disease?**

Opening question: to provide me a picture of your and your child’s situation, can you briefly tell me how things have gone with …. (name child) from the moment it became known that he/she is ill?

- What do you consider to be your responsibilities as a parent for your ill child?

Perception of your own situation / child’s situation

- How is your child doing at this moment?
- What kind of care and caregiving does your child need (from you and others)?
- Can you describe the caregiving for your child on any given day in the past week?
- What does the caregiving require from you?

Confering Meaning

- What is it like for you to care for a seriously ill child?
- Are there things that are difficult for you in the caregiving for your child? What makes them difficult?
- What concerns you the most at this moment?

Being a parent caring for a seriously ill child:

- What do you think are the most important things you do for your child?
- What can you leave to others?
- What can you absolutely not leave to others?
- Are there aspects of your support for your child that you feel uncomfortable about or that you have difficulties with? And are there aspects that are rewarding or that gives you a good feeling?
- What do you expect for the future?

Impact on the parents’ and siblings’ lives:

- What does the caregiving for your child mean for the functioning of your daily life?
- What does the caregiving for your child mean for the functioning of your family?
- To what extent does the illness of your child affect your ability to raise your child?
- How do you think about raising your child?
- To what extent do you care for your child together with your partner?

**What are the demands and stressors parents have to deal with as a result of the caregiving for their ill child?**

Coping, self-efficacy

- Some parents feel they can deal with their situation, other parents have the feeling they are collapsing under the strain. What is it like for you?
  - How do you see your ability to cope in the future?
- What would help you to cope better with your situation?

Palliative care at home

- Some parents care for their child at home, others make use of care facilities, such as a daycare or a hospice, how is your child’s situation?
- How was this care decision made?
  - What considerations were taken into account?
- Are you aware that there are other possibilities? / Have you ever considered doing otherwise?

Aims

Professional care often works with goals. Parents often ‘just seem to do it’/’just seem to care for their child’ but you might also have some aims for your child/family that you are striving for?

Crisis management

- Have there been moments when you thought: I just don’t know what to do anymore? Have you taken any action on that basis?
- What events give you the feeling that you are out of balance?
- What do you think might happen in the future that would make you lose your balance?
- Who or what supports you in such situations?
